# Pain and Function Recovery Trajectories following Revision Hip Arthroplasty: Short-Term Changes and Comparison with Primary Hip Arthroplasty in the ADAPT Cohort Study

**DOI:** 10.1371/journal.pone.0164839

**Published:** 2016-10-14

**Authors:** Erik Lenguerrand, Michael R. Whitehouse, Vikki Wylde, Rachael Gooberman-Hill, Ashley W. Blom

**Affiliations:** Musculoskeletal Research Unit, School of Clinical Sciences, University of Bristol, Level 1 Learning and Research Building, Southmead Hospital, Westbury-on-Trym, Bristol, BS10 5NB, United Kingdom; University of Brescia, ITALY

## Abstract

**Background and Purpose:**

Patients report similar or better pain and function before revision hip arthroplasty than before primary arthroplasty but worse results are reported after revision surgery than after primary surgery. The trajectory of post-operative recovery during the first months and any differences by type of surgery have received little attention. We explored the trajectories of change in pain and function after revision hip arthroplasty to 12-months post-operatively and compare them with those observed after primary hip arthroplasty.

**Methods:**

This study is a prospective cohort study of patients undergoing primary (n = 80 with 92% for an indication of osteoarthritis) and revision (n = 43) hip arthroplasties. WOMAC pain and function scores and walking speed were collected pre-operatively, at 3 and 12-months post-operatively. Multilevel regression models were used to chart and compare the trajectories of change (0–3 months and 3–12 months) between types of surgery.

**Results:**

The improvements in pain and function following revision arthroplasty occurred within the first 3-months with no evidence of further change beyond this initial period. While the pattern of recovery was similar to the one observed after primary arthroplasty, improvements in the first 3-months were smaller after revision compared to primary arthroplasty. Patients listed for revision surgery reported lower pre-operative pain levels but similar post-operative levels compared to those undergoing primary surgery. At 12-months post-operation patients who underwent a revision arthroplasty had not reached the same level of function achieved by those who underwent primary arthroplasty.

**Conclusion:**

The post-operative improvements in pain and function are larger following primary hip arthroplasty than following revision hip arthroplasty. Irrespectively of surgery type, most of the improvements occur in the first three post-operative months. More research is required to identify whether the recovery following revision surgery could be improved with specific post-operative interventions.

## Introduction

The volume of primary hip arthroplasties rose by approximately 26% between 2010 and 2015 [1, 2]. Over 88,000 primary total hip arthroplasties are performed per year in England and Wales [2]. These figures will continue to rise due to increases in obesity and an aging community [3]. The revision burden is approximately 11% and over 9,500 revision hip arthroplasties were performed in England and Wales in 2015 [1, 2].

Both primary and revision hip arthroplasty have been shown to improve patient-reported pain and function for the majority of patients [4–8]. While patients tend to have similar [5, 6, 8] or better [7] pain and function prior to revision arthroplasty than prior to primary arthroplasty, patients who undergo primary surgery report better post-operative outcomes than those who undergo revision surgery [5–7].

Typically, the outcome of both primary and revision is reported at 12-months or more after surgery [6–8]. The pattern of recovery trajectories within the first 12-months after surgery, and differences between primary and revision surgery in this period have received little attention. Pain and function have previously been reported not to improve further after 6-months following revision arthroplasty [9, 10]. In the absence of assessment prior to 6-months in these studies the pattern of improvement in the first months post-operation requires further investigation.

Evidence from the ADAPT cohort study suggests that in primary hip arthroplasty, the improvement in patient-reported pain and function post-operation plateaus at 3-months [11]. It is not currently clear if the pattern of recovery following revision hip arthroplasty is similar or if the complexity, extent of surgery and surgical trauma leads to a different pattern.

To describe and explore potential disparities in the degree and pattern of post-operative recovery following revision hip arthroplasty, we analysed data collected pre-operatively, at 3- and 12-months post-operation from the ADAPT prospective cohort study. Specifically our research aims were 1. to describe the early trajectories of pain and function after revision hip arthroplasty, 2. compare these trajectories with those observed after primary hip arthroplasty and 3. compare the post-operative outcomes achieved after these two types of surgery. We also investigated whether the pattern of recovery was similar when function is objectively assessed with standardised performance tests compared to patient-reported outcome measures.”

## Materials and Methods

This study followed the STROBE (STrengthening the Reporting of OBservational studies in Epidemiology) guidelines for reporting observational studies in epidemiology (Appendix A in S1 File).

### Study design

ADAPT is a single-centre UK prospective cohort study including patients undergoing hip or knee arthroplasty (UKCRN ID 8311). National Health Service Research Ethics Committee approval was granted for the study (09/H0102/72) and all patients provided informed, written consent.

Detailed information on study design, patient recruitment, inclusion-exclusion criteria, and assessment methods are provided in the published study protocol [12]. Briefly, between February 2010 and November 2011, patients waiting for hip or knee arthroplasty at a high-volume elective orthopaedic centre were invited to participate in the study. Approximately 250 patients were recruited to ensure a sufficient number of patients to perform meaningful data analysis. Patients were due to undergo a range of primary and revision arthroplasty procedures (primary total knee arthroplasty, unicompartmental knee arthroplasty, patellofemoral arthroplasty, revision total knee arthroplasty, primary total hip arthroplasty, primary hip resurfacing or revision total hip arthroplasty) so that functional measures could be investigated across a range of patients with diverse indications for surgery and degrees of functional impairment. The majority of patients listed for primary arthroplasty had an indication of osteoarthritis. Exclusion criteria included an inability to provide written informed consent, to complete English language questionnaires (not all the questionnaires we used have been translated or validated for use in other languages), participation to another study, and severe functional limitations which would prevent completion of a performance test. In particular, patients using wheelchairs were excluded.

This analysis was restricted to patients who underwent primary total, resurfacing or revision hip arthroplasty.

### Data collection

Assessments were conducted before surgery (median 19 days) and then at 3 and 12-months after surgery. At each post-operative assessment time, participants completed a postal questionnaire.

### Participant and surgical characteristics

Data on gender, age, living arrangement, level of education, working status and number of joints affected by arthritis were collected in the pre-operative questionnaire. The indication for surgery, type of surgery, surgical approach, height and weight were extracted from participants’ medical records.

Patients undergoing primary arthroplasty had a total hip replacement (n = 74) or hip resurfacing (n = 6). Osteoarthritis was the indication for surgery in 92% of primary cases. Patients undergoing revision arthroplasty had revision of a total hip arthroplasty (86%, n = 37), hip resurfacing (9%, n = 4), or hemiarthroplasty (5%, n = 2). The most common indication for revision arthroplasty was aseptic loosening (67%, n = 29); the remaining indications were pain (9%, n = 4), aseptic lymphocyte-dominated vasculitis-associated lesion (9%, n = 4) and other reasons (11%, n = 6). Primary (87%, n = 70) and revision arthroplasties (98%, n = 42) were mostly commonly performed via a posterior surgical approach.

### Patient-reported measures

Self-reported pain and function were assessed using the Western Ontario and McMaster Universities Osteoarthritis Index (WOMAC) function and pain sub-scales [13]. The WOMAC-function measure consists of 17 questions assessing the extent of functional limitation when performing a range of daily activities. WOMAC-pain consists of five questions assessing pain during walking, using stairs, in bed, sitting or lying. Each sub-score ranges from 0–100 (worst to best). The WOMAC score has good psychometric properties with test-retest reliability above 0.8 for the physical function subscale and above 0.7 for the pain subscale [14].

### Performance test

An objective measure of function was obtained using a timed walk test [15]. Participants were timed and supervised by a research nurse as they walked a 20 metres straight distance on level ground at their normal, comfortable speed. Speed (metres per second) was derived by dividing the distance walked by the time required to complete the task. The test-retest reliability of the 20 metres has been showed to be high (> 0.9) [16, 17].

### Statistical analysis

Three random intercept and slope linear regression models, one for each studied outcome (WOMAC-pain, WOMAC-function and walking speed), were used to investigate the pattern of post-operative changes following revision hip arthroplasty (aim 1) and compare the changes with those following primary hip arthroplasty (aim 2). This approach accounts for repeated and unequal numbers of measurements per participant while producing estimations valid under the missing at random assumption [18]. In this modelling framework, all available pre- and post-operative assessments of the outcome of interest were modelled. The outcomes were standardised (using the pre-operative mean and standard deviation of the score of interest) to produce estimates comparable across models. Those outcomes were regressed on an intercept (mean of standardised outcome on day of surgery at the sample mean age), age (centred at 65.2, the overall sample mean age) and two time splines (with random effect on their associated effects): one spline (a line between two points) for the “short-term change” occurring between the pre-operative assessment and the second assessment (3-months post-operative) and another spline for the “long-term change” occurring between the two post-operative assessments (3 and 12-months). Changes between assessment points were modelled rather than the actual scores achieved at 3- or 12-months. This was because the distributions of the scores were strongly skewed but the changes were normally distributed and could be analysed with the model framework presented above (as evidenced by the residuals plots). These models were stratified by primary/revision status to produce estimates specific to each type of surgery. Comparisons of the short- and long-term changes by surgery type were performed using their fixed effects and contrasts.

The equation structure of these models is described in more details in appendix B in S1 File with the code used to compute them.

The short- and long-term changes were also plotted by surgery type. For this purpose, the random intercept and slope linear model framework described above was re-run unadjusted for age on the unstandardised outcomes of pain and function. The fixed effects associated with the intercepts, and time splines of the primary and revision arthroplasty equations were used to produce the mean changes and their 95%CI.

Finally, the post-operative outcomes achieved at 3- and 12-months post-operatively were compared by surgery type (aim 3). As explained, the actual post-operative outcomes were strongly skewed and could not be investigated within the regression framework. Mann-Whitney tests were used for this purpose.

All models were fitted using Stata SE 13.1 (StataCorp LP, College Station, Texas, USA) and MLwiN v2.31 using Stata runmlwin command [19]. A p-value of <0.05 was considered as evidence of statistical significance.

## Results

### Sample description

**Fig 1:**
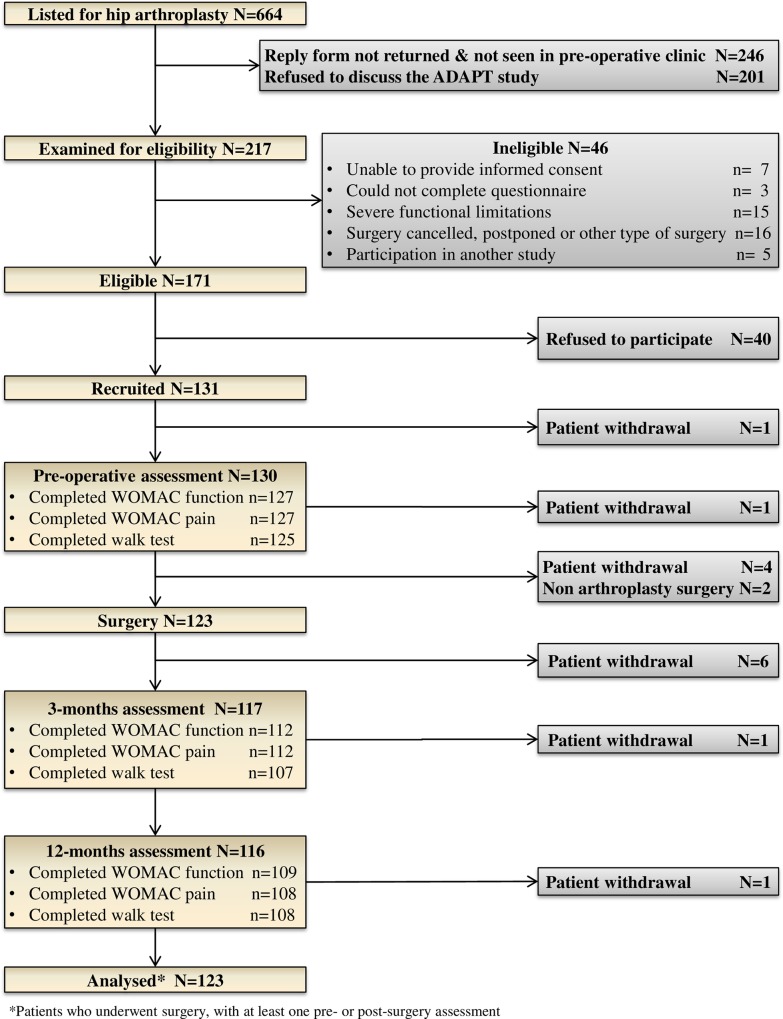
Flow diagram of recruitment and participation.

Overall, 664 patients were identified on the waiting list for primary or revision hip arthroplasty (Fig 1). A total of 447 patients were not approached or refused to discuss the study. Forty-six patients were also ineligible among which 15 were wheelchair users including two with severe balance issue. A total of 171patients were eligible and 131 agreed to take part (77%). Eight patients did not subsequently undergo hip arthroplasty and therefore 123 patients were included in the final analysis. Of these patients 80 had a primary and 43 had a revision hip arthroplasty.

All these 123 participants had at least one assessment (pre-operative, 3 and/or 12-months) for any of the investigated measures (WOMAC-pain, WOMAC-function scores or walking speed) and were considered in the analyses. A description of the available number of assessments at each data collection points is provided in Table 1. The percentage of participants with complete information on WOMAC-pain, WOMAC-function scores and walking speed at 12-months post-operation was comparable between type of surgery (89% and 84% for primary and revision arthroplasty respectively).

**Table 1:** Pain and function by assessment period and revision/primary profile.

N = 123	Pre-operative			3-months			12-months		
	Total	Primary	Revision	Total	Primary	Revision	Total	Primary	Revision
**WOMAC Pain**	121	78	43	112	76	36	108	71	37
missing, n =	2	2	0	11	4	7	15	9	6
%	98.4	64.5	35.5	91.1	67.9	32.1	87.8	65.7	34.3
Median	55	55	60	95	95	95	100	100	95
Interquartile range^a^	[35, 70]	[30, 70]	[50, 75]	[80, 100]	[85, 100]	[73, 100]	[85, 100]	[90, 100]	[80, 100]
p-value^b^	0.031			0.479			0.268		
**WOMAC function**	121	78	43	112	76	36	109	71	38
missing, n =	2	2	0	11	4	7	14	9	5
%	98.4	64.5	35.5	91.1	67.9	32.1	88.6	65.1	34.9
Median	56	54	62	90	90	89	94	96	93
Interquartile range^a^	[38, 71]	[38, 71]	[41, 75]	[81, 96]	[81, 96]	[79, 96]	[84, 99]	[87, 100]	[76, 97]
p-value^b^	0.165			0.678			0.015		
**Walking-speed**^**c**^	118	77	41	107	74	33	108	72	36
missing, n =	5	3	2	16	6	10	15	8	7
%	95.9	65.3	34.8	87	69.2	30.8	87.8	66.7	33.3
Median	0.91	0.91	0.83	1.11	1.11	1.05	1.18	1.18	1.11
Interquartile range^a^	[0.71, 1.11]	[0.71, 1.11]	[0.67, 1.11]	[0.91, 1.25]	[0.95, 1.25]	[0.91, 1.18]	[0.95, 1.33]	[1.03, 1.38]	[0.87, 1.18]
p-value^b^	0.464			0.343			0.004		

a First and third quartiles: 25^th^ and 75^th^ percentiles

b Mann-Whitney test to compare median scores by primary/revision profile.

c Walking-speed expressed in metres per second: 20 metres / Completion-time

The characteristics of the cohort are shown in Table 2. The mean age was 65 years (SD 11) and 66 years (SD 11) respectively for participants who underwent a primary and revision arthroplasty respectively. The median body mass index was 26 kg/m^2^ (Interquartile range (IQR) 24–29) and 28 (24–28) respectively.

**Table 2:** Participant characteristics.

				Primary		Revision	
		N = 123	%	n = 80	%	n = 43	%
**Sex**	Men	61	49.6	38	47.5	23	53.5
	Women	62	50.4	42	52.5	20	46.5
**Number of other joints with OA**	None	25	20.3	20	25.0	5	11.6
	One joint	29	23.6	22	27.4	7	16.3
	Two joints	22	17.9	13	16.3	9	20.9
	3 joints	18	14.6	8	10.0	10	23.3
	> = 4 joints	22	17.9	13	16.3	9	20.9
	Unknown	7	5.7	4	5.0	3	7.0
**Living alone**	Living with someone	90	73.2	59	73.7	31	72.1
	Living alone	30	24.4	18	22.5	12	27.9
	Unknown	3	2.4	3	3.8	0	0.0
**Education**	Normal school leaving age	66	53.7	41	51.2	25	58.1
	College	26	21.1	20	25.0	6	14.0
	University	28	22.8	16	20.0	12	27.9
	Unknown	3	2.4	3	3.8	0	0.0
**Working status**	Full time	55	44.7	34	42.5	21	48.8
	Retired	60	48.8	38	47.5	22	51.2
	Unemployed	7	5.7	7	8.7	0	0.0
** **	Unknown	1	0.8	1	1.3	0	0.0

### Pain and function trajectories after revision arthroplasty

**Fig 2:**
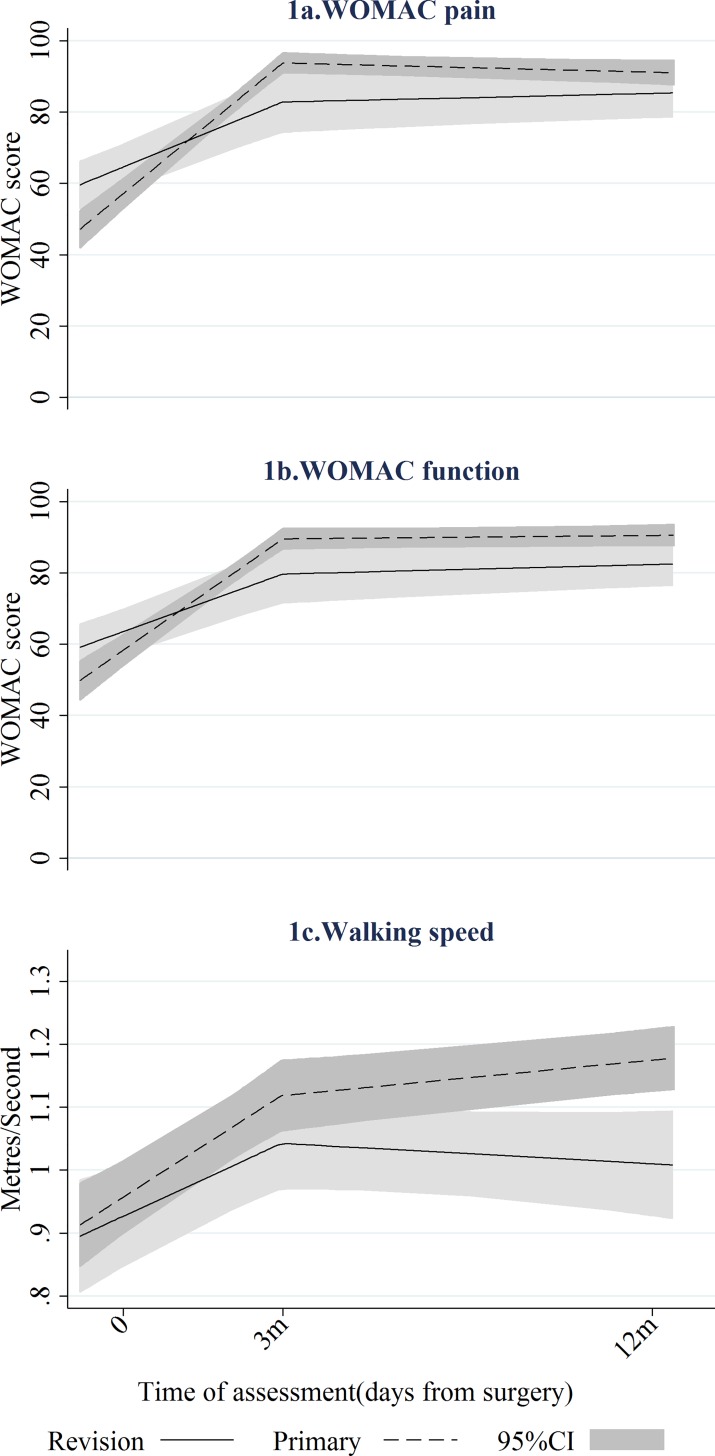
Mean trajectories^a^ for WOMAC-pain, WOMAC-function and walking speed (Unstandardised outcomes) by revision/primary surgery. The mean trajectories are derived using the fixed effects terms of the linear mixed models stratified on primary-revision profile and regressing each outcome on the time of assessment parameterised as two linear splines.

Revision hip arthroplasty lead to a significant improvement in both pain and function (Fig 2). Changes in pain and function occurred within the first 3-months post-operation (WOMAC-pain, p <0.0001; WOMAC-function, p<0.0001; Walking speed, p<0.0001; S1 Table). No evidence of further improvement in pain or function was found between 3 and 12-months (S1 Table). Pain and function trajectories after primary arthroplasty are reported in Fig 2 and S1 Table and have been previously described [11]. Changes mainly occur within the first 3-months following surgery and there is no evidence of further changes after 3-months with the exception of the walking speed which continued to marginally improved between 3 and 12-months (p = 0.005).

### Comparisons between revision and primary arthroplasty

Pre-operatively, the level of pain reported by participants listed for primary surgery was worse than for those listed for revision surgery (median 55 vs. 66, p = 0.031, Table 1). However greater short-term improvements in WOMAC-pain (Fig 2A) were assessed during the first 3-months following primary arthroplasty compare to the changes found after revision arthroplasty (p<0.0001, S1 Table). No evidence of change was found between 3 and 12-months for either type of surgery. As a result there was no more significant difference at 3 (p = 0.479, Table 1) or 12-months (p = 0.268, Table 1).Pre-operatively, the median WOMAC-function scores were not different between those that underwent primary and revision hip arthroplasty (Table 1). The mean short-term change in the WOMAC-function score (Fig 2B) following revision arthroplasty was smaller than that following primary arthroplasty (p<0.001, S1 Table). No evidence of long-term change (between 3 and 12-months) was observed for either type of surgery. At 12-months post-operation, the median WOMAC-function score was higher after primary surgery than after revision surgery (96 vs 93, p = 0.015, Table 1).

The walking speed was comparable pre-operatively for participants who subsequently underwent primary and revision arthroplasty (p = 0.464, Table 1). The trajectory of recovery exhibited by the walking speed differed between patients with revision and primary arthroplasty (Fig 2C). The speed improved to a similar extent during the first 3-months following both types of surgery. However, the improvement continued after 3-months for primary arthroplasty (p = 0.005, S1 Table) but not for revision arthroplasty (p = 0.300, S1 Table). Participants who had undergone revision arthroplasty reported a slower walking speed at 12-months post-operatively than those who had undergone primary (p = 0.004; Table 1).

## Discussion

Investigation of the early outcome trajectories after revision hip arthroplasty has revealed that the improvements in pain and function were mainly observed within the first 3 post-operative months with no evidence of further change beyond this initial period. The comparisons of these trajectories with those observed after primary hip arthroplasty have shown that while pain or function recovery was plateauing before six-months for both surgery types, the extent of improvements was different with smaller short-term changes after revision arthroplasty than after primary arthroplasty. Comparisons of the achieved post-operative outcomes reveal that patients who underwent revision surgery reported less pre-operative pain than those who underwent primary surgery but due to the difference in the extent of post-operative changes this advantage was not sustained post-operatively. While function was comparable pre-operatively, at 12-months post-operation patients who underwent revision arthroplasty had not reached the level of function achieved by those who underwent primary arthroplasty. Finally, some difference in the pattern of recovery was observed when function was objectively assessed. Contrary to patient-reported function, minor but statistically significant improvements in walking-speed was observed between 3 and 12-months after primary arthroplasty and this long-term changes were not observed after revision surgery.

The observed effectiveness of revision hip arthroplasty to improve patient-reported pain and function is consistent with the existing evidence [9, 20–25]. The few studies measuring outcomes prior to 12 months post-operation report that changes in outcomes following revision hip arthroplasty plateau at 6-months post-operation [9, 10]. None of these studies has measured pain or function at 3-months post-operation and the current study filled this gap. This “plateau” was reached at least 3-months earlier than previously shown and was lower than the one observed after primary arthroplasty. This suggests that the higher complexity or degree of trauma related to revision surgery as compared to primary surgery limits the extent of the recovery but does not increase the time taken to recover: patients undergoing revision arthroplasty will improve but should not expect to achieve outcomes as high as those reached after their primary surgery.

The differential between the degree of improvement following revision and/or the outcome level reached post-operatively compared to primary surgery have also been shown but only in the post-operative period starting 12 months or more after surgery [4–8, 25–28]. In this respect, the current findings fill another gap in the literature.

There is limited evidence on the improvement in objective function following revision hip arthroplasty but the findings are in agreement with ours[20]. Aghayev et al. demonstrated the benefit of revision hip arthroplasty on the ability to walk, reporting an improvement in the percentage of their patients unable to walk for more than 30 minutes 12-months after surgery from 65% pre-operatively to 50% 12-months after surgery. Similar to our observations, the improvement was less than that observed following primary surgery.

The strengths of this study are the availability of patient reported outcomes measured at 3-months post-operation in addition to objective function assessment with a performance test. Using an objective measurement allows us to ascertain that the lack of functional improvement beyond 3-months among the patients who underwent a revision surgery is not due to the inherent ceiling effect associated with using a score-bounded patient-reported outcome such as the WOMAC score [29–32]: the lack of long-term improvement was also observed when function was measured with the walking speed, an objective tool that may be less subject to ceiling-effect. Moreover, among patients undergoing primary surgery, the mean long-term improvement of objective function was significant but small compared to the short-term mean improvement (0.02 vs 0.15, Table 2), confirming that most of the functional changes, whether objectively or subjectively measured, occur before the 3-month post-operative time point.

This study is not without limitations. The findings were obtained on patients from a single-centre orthopaedic unit limiting their external validity. The modest sample size restricted our ability to adjust for factors known to be associated with post-operative outcomes such as gender, mental health and co-morbidities [26, 33, 34]. However, our findings were adjusted for age. A larger sample would nevertheless have been required to adjust for additional confounding factors, in particular type or indication for surgery. As all patients undergoing revision surgery and 93% of those undergoing primary surgery received a total hip replacement, our comparisons between revision and primary arthroplasty are more generalizable to patients undergoing total joint replacement. The remaining 7% (n = 6) of patients undergoing primary arthroplasty were listed for resurfacing surgery. While they exhibit comparable pre- and post-operative functional outcomes their pain at 12 months post-operation was worse than for those listed for primary total arthroplasty (Medians: 75 vs 100; p-values = 0.03). The group of patients undergoing revision surgery was modest in size (n = 43) but relatively homogeneous with 86% being revision of a primary total arthroplasty rather than after a previous revision. A larger revision group would have allowed the stratification of the analysis by indication for surgery. The post-operative outcomes following revision arthroplasty have been shown to be influenced by the indication for surgery [22, 35] and therefore our findings are more reflective of those revised for aseptic loosening (>67% of the revised participants). No information on the pre- and post-operative treatment received by the participants was available. They were offered standard care as provided at the treating centre. This comprised a pre-operative educational class focusing on preparation for surgery and the hospital stay, and post-operative outpatient physiotherapy on a needs basis. Finally, the inclusion of additional assessment points prior to 3-months would have allowed more detailed investigation of the very early recovery trajectories. We considered that additional assessment points would have represented an excess burden for participants with a probability of increased levels of attrition in the cohort.

## Conclusions

Patients undergoing revision hip arthroplasty should be informed that the expected improvement following such surgery will be less marked than that expected and experienced for primary surgery and the majority of their improvement will occur in the first 3 post-operative months.

More research is now required to identify whether specific in-patient and post-discharge rehabilitation tailored towards patients undergoing revision arthroplasty would improve or achieve equivalent outcomes to primary surgery and whether patients who are achieving limited improvements at 3-months post-operative would beneficiate from longer or more intensive rehabilitation. This will become all the more important with the increasing volume of revision surgery and the high expectations of patients who aspire to a disease-free and active life [36–38].

## Supporting Information

S1 File Appendices. (DOCX)

S1 Table Univariate age-adjusted linear mixed regression models of function and pain stratified by revision/primary profiles. (DOCX)
