# Supplementary material for: Pain and Function Recovery Trajectories following Revision Hip Arthroplasty: Short-Term Changes and Comparison with Primary Hip Arthroplasty in the ADAPT Cohort Study
Source: PLoS One. 2016 Oct 14;11(10):e0164839. doi: 10.1371/journal.pone.0164839 (PMC5065160; doi:10.1371/journal.pone.0164839)
Supplement: S1 File — (DOCX) [file pone.0164839.s001.docx]

**Appendix A: STROBE Statement**

|  | Item No | Recommendation | Action |
| --- | --- | --- | --- |
| **Title and abstract** | 1 | (*a*) Indicate the study’s design with a commonly used term in the title or the abstract | Design described in title and abstract |
|  |  | (*b*) Provide in the abstract an informative and balanced summary of what was done and what was found | Done |
| Introduction | | |  |
| Background/rationale | 2 | Explain the scientific background and rationale for the investigation being reported | Done |
| Objectives | 3 | State specific objectives, including any prespecified hypotheses | Done |
| Methods | | |  |
| Study design | 4 | Present key elements of study design early in the paper | See Study design section of Methods |
| Setting | 5 | Describe the setting, locations, and relevant dates, including periods of recruitment, exposure, follow-up, and data collection | See Study design section of Methods |
| Participants | 6 | (*a*) Give the eligibility criteria, and the sources and methods of selection of participants. Describe methods of follow-up | See Study design and Data collection sections of Methods. More information also available here  http://bmcmusculoskeletdisord.biomedcentral.com/articles/10.1186/1471-2474-13-220 |
|  |  | (*b*) For matched studies, give matching criteria and number of exposed and unexposed | Not applicable |
| Variables | 7 | Clearly define all outcomes, exposures, predictors, potential confounders, and effect modifiers. Give diagnostic criteria, if applicable | See Participant and surgical characteristics, Patient-reported measures and Performance test sections of Methods |
| Data sources/ measurement | 8* | For each variable of interest, give sources of data and details of methods of assessment (measurement). Describe comparability of assessment methods if there is more than one group | See Participant and surgical characteristics, Patient-reported measures and Performance test sections of Methods |
| Bias | 9 | Describe any efforts to address potential sources of bias | See Statistical analysis section of Methods |
| Study size | 10 | Explain how the study size was arrived at | See Study design section of Methods |
| Quantitative variables | 11 | Explain how quantitative variables were handled in the analyses. If applicable, describe which groupings were chosen and why | See Statistical analysis section of Methods |
| Statistical methods | 12 | (*a*) Describe all statistical methods, including those used to control for confounding | See Statistical analysis section of Methods |
|  |  | (*b*) Describe any methods used to examine subgroups and interactions | See Statistical analysis section of Methods and Appendix 2 |
|  |  | (*c*) Explain how missing data were addressed | See Statistical analysis section of Methods |
|  |  | (*d*) If applicable, explain how loss to follow-up was addressed | See Statistical analysis section of Methods |
|  |  | (*e*) Describe any sensitivity analyses | Not applicable |
| Results | | |  |
| Participants | 13* | (a) Report numbers of individuals at each stage of study—eg numbers potentially eligible, examined for eligibility, confirmed eligible, included in the study, completing follow-up, and analysed | See figure 1 and beginning of results section |
|  |  | (b) Give reasons for non-participation at each stage | See figure 1 |
|  |  | (c) Consider use of a flow diagram | See figure 1 |
| Descriptive data | 14* | (a) Give characteristics of study participants (eg demographic, clinical, social) and information on exposures and potential confounders | See table 2 |
|  |  | (b) Indicate number of participants with missing data for each variable of interest | See tables 1 and 2 |
|  |  | (c) Summarise follow-up time (eg, average and total amount) | See figures |
| Outcome data | 15* | Report numbers of outcome events or summary measures over time | See table 1 |
| Main results | 16 | (*a*) Give unadjusted estimates and, if applicable, confounder-adjusted estimates and their precision (eg, 95% confidence interval). Make clear which confounders were adjusted for and why they were included | See appendix table 1 |
|  |  | (*b*) Report category boundaries when continuous variables were categorized | See tables |
|  |  | (*c*) If relevant, consider translating estimates of relative risk into absolute risk for a meaningful time period | Not aplicable |
| Other analyses | 17 | Report other analyses done—eg analyses of subgroups and interactions, and sensitivity analyses | See Comparisons between revision and primary arthroplasty section of Results |
| Discussion | | |  |
| Key results | 18 | Summarise key results with reference to study objectives | See first section of Discussion |
| Limitations | 19 | Discuss limitations of the study, taking into account sources of potential bias or imprecision. Discuss both direction and magnitude of any potential bias | See last section of Discussion |
| Interpretation | 20 | Give a cautious overall interpretation of results considering objectives, limitations, multiplicity of analyses, results from similar studies, and other relevant evidence | See Discussion and Conclusions |
| Generalisability | 21 | Discuss the generalisability (external validity) of the study results | See last section of Discussion |
| Other information | | |  |
| Funding | 22 | Give the source of funding and the role of the funders for the present study and, if applicable, for the original study on which the present article is based | Describe during online submission process and see Acknowledgments and funding |

*Give information separately for exposed and unexposed groups.

**Note:** An Explanation and Elaboration article discusses each checklist item and gives methodological background and published examples of transparent reporting. The STROBE checklist is best used in conjunction with this article (freely available on the Web sites of PLoS Medicine at http://www.plosmedicine.org/, Annals of Internal Medicine at http://www.annals.org/, and Epidemiology at http://www.epidem.com/). Information on the STROBE Initiative is available at http://www.strobe-statement.org.

**Appendix B: Equation structure and Stata code for the random intercept and slope linear model stratified by surgery profile**

The equation for a random intercept and slope linear model stratified by surgery profile is as follows:

$\text{y}_{\text{ij}}\text{=}\text{β}_{\text{0}}\text{+u}_{\text{0j}}\text{+β}_{\text{1}}\text{ . age}+\text{ I}_{\left( \text{Measurement}_{\text{ij}}\text{<3} \right)}\text{ . [}\left( \text{β}_{\text{2}}\text{+u}_{\text{2j}} \right)\text{.}\text{Measurement}_{\text{ij}}\text{ ] +}\text{ (1-I}_{\left( \text{Measurement}_{\text{ij}}\text{<3} \right)}\text{) . [}\left( \text{β}_{\text{2}}\text{+u}_{\text{2j}} \right) \text{.} 3+ \left( \text{β}_{3}\text{+u}_{\text{3j}} \right) \text{.}(\text{Measurement}_{\text{ij}}-3)]$ if surgery=revision arthroplasty

or

$\text{y}_{\text{ij}}\text{=}\text{β}_{4}\text{+u}_{\text{4j}}\text{+β}_{\text{5}}\text{ . age}+\text{ I}_{\left( \text{Measurement}_{\text{ij}}\text{<3} \right)}\text{ . [}\left( \text{β}_{\text{6}}\text{+u}_{\text{6j}} \right)\text{.}\text{Measurement}_{\text{ij}}\text{ ] +}\text{ (1-I}_{\left( \text{Measurement}_{\text{ij}}\text{<3} \right)}\text{) . [}\left( \text{β}_{\text{6}}\text{+u}_{\text{6j}} \right) \text{.} 3+ \left( \text{β}_{\text{7}}\text{+u}_{\text{7j}} \right) \text{.}(\text{Measurement}_{\text{ij}}-3)]$ if surgery=primary arthroplasty

With $\left[ \begin{matrix} \text{u}_{\text{0j}} \\ \text{u}_{\text{2j}} \\ \text{u}_{\text{3j}} \\ \text{u}_{\text{4j}} \\ \text{u}_{\text{6j}} \\ \text{u}_{\text{7j}} \end{matrix} \right]$~ N(0, $\Omega_{\text{u}}$): $\Omega_{\text{u}}=\left[ \begin{matrix} \sigma_{u_{0}}^{2} & & & & & \\ \sigma_{u_{02}} & \sigma_{u_{2}}^{2} & & & & \\ \sigma_{u_{03}} & \sigma_{u_{23}} & \sigma_{u_{3}}^{2} & & & \\ 0 & 0 & 0 & \sigma_{u_{4}}^{2} & & \\ 0 & 0 & 0 & \sigma_{u_{46}} & \sigma_{u_{6}}^{2} & \\ 0 & 0 & 0 & \sigma_{u_{47}} & \sigma_{u_{67}} & \sigma_{u_{7}}^{2} \end{matrix} \right]$

Where

- y*_ij_*  ~ N(XB, Ω) is the outcome of interest (pain or function) measured at assessment i (i=pre-operation, 3-months and 12-months visit) on participant j
- $\text{Measurement}_{\text{ij}}$ is the time of the outcome assessment i performed on participant j centred on the date of surgery and expressed in months
- $\text{β}_{\text{0}}$ and $\text{β}_{\text{4}}$ are respectively the fixed coefficients describing the intercept in the revision, primary arthroplasty group, i.e. the mean outcome value when $\text{Measurement}_{\text{ij}}=0$ - at the time of surgery- and age is at the overall sample mean age
- $\text{β}_{\text{1}}$ and $\text{β}_{\text{5}}$ are the fixed coefficients describing the average effect of participant age in the revision and primary arthroplasty groups. Age is centred at the overall sample mean age.
- $\text{ I}_{\left( \text{Measurement}_{\text{ij}}\text{<3} \right)}$ is an indicator function equal to 1 if the participant outcome was assessed before 3 months and 0 otherwise.
- $\text{β}_{\text{2}}$ and $\text{β}_{\text{6}}$ are the fixed coefficients describing the average slope between the baseline and 3-months assessments in the revision and primary arthroplasty groups, i.e the spline for the short-term changes
- $\text{β}_{\text{3}}$ and $\text{β}_{\text{7}}$ are the fixed coefficients describing the average slope between the 3- and 12-months assessments in the revision and primary arthroplasty groups, i.e the spline for the long-term changes
- $\text{u}_{\text{0}}$ and $\text{u}_{\text{4}}$ are the random coefficients describing the departure of participant j from the average intercept in the revision and primary arthroplasty groups.
- $\text{u}_{\text{2}}$ and $\text{u}_{\text{6}}$ are the random coefficients describing the departure of participant j from the average short-term changes slope in the revision and primary arthroplasty groups.
- $\text{u}_{\text{3}}$ and $\text{u}_{\text{7}}$ are the random coefficients describing the departure of participant j from the average long-term changes slope in the revision and primary arthroplasty groups.

**Stata code:**

gen t1 = Assessment_time

replace t1 = 3 if Assessment_time >=3

gen t2 = (Assessment_time - 3 )

replace t2 = 0 if Assessment_time <3

by study_id: gen obs=_n

sort study_id obs

gen cons_revision = 1 if revision==1

gen t1_revision =t1 if revision==1

gen t2_revision =t2 if revision==1

replace cons_revision = 0 if revision==0

replace t1_revision =0 if revision==0

replace t2_revision =0 if revision==0

gen cons_primary= 1 if revision==0

gen t1_ primary =t1 if revision==0

gen t2_ primary =t2 if revision==0

replace cons_primary = 0 if revision==1

replace t1_ primary =0 if revision==1

replace t2_ primary =0 if revision==1

matrix A = (1,1,1,1,1,1,0,0,0,1,0,0,0,1,1,0,0,0,1,1,1)

*****Unadjusted for age

capture drop U0 U1 U2 U3 U4 U5 U0se U1se U2se U3se U4se U5se

runmlwin y cons_revision t1_revision t2_revision cons_primary t1_ primary t2_revision, sd level1(obs: , ) level2(study_id: cons_revision t1_revision t2_revision cons_primary t1_ primary t2_revision , residual( U) elements(A) ) nopause

*****Adjusted for age

gen age_revision = Age-65.2 if revision==1 /*centred on sample mean age*/

replace age_revision = 0 if revision==0

gen age_primary = Age-65.2 if revision==0 /*centred on sample mean age*/

replace age_primary = 0 if revision==1

runmlwin y cons_revision t1_revision t2_revision age_revision cons_primary t1_ primary t2_revision age_primary , sd level1(obs: , ) level2(study_id: cons_revision t1_revision t2_revision cons_primary t1_ primary t2_revision , residual( U) elements(A) ) nopause

***test of the difference between the revision and primary short-term mean changes

lincom t1_revision-t1_primary

***test of the difference between the revision and primary long-term mean changes

lincom t2_revision-t2_primary
