# Supplementary material for: Pain and Function Recovery Trajectories following Revision Hip Arthroplasty: Short-Term Changes and Comparison with Primary Hip Arthroplasty in the ADAPT Cohort Study
Source: PLoS One. 2016 Oct 14;11(10):e0164839. doi: 10.1371/journal.pone.0164839 (PMC5065160; doi:10.1371/journal.pone.0164839)
Supplement: S1 Table — (DOCX) [file pone.0164839.s002.docx]

**S1 Table. Univariate age-adjusted linear mixed regression models of function and pain stratified by revision/primary profiles.**

|  | **Revision surgery** | | | | **Primary surgery** | | | | **Revision vs. Primary^d^** | | | |
| --- | --- | --- | --- | --- | --- | --- | --- | --- | --- | --- | --- | --- |
|  | **Coef.** | **[95% Conf. Interval]** | | **p-value** | **Coef.** | **[95% Conf. Interval]** | | **p-value** | **Difference** | **[95% Conf. Interval]** | | **p-value** |
| **WOMAC-pain** |  |  |  |  |  |  |  |  |  |  |  |  |
| Pre-operative score^a^ | **0.490** | (0.201, | 0.778) | 0.001 | 0.149 | (-0.040, | 0.338) | 0.123 | 0.341 | (-0.004, | 0.686) | 0.053 |
| Short-term change^b^ | **0.228** | (0.155, | 0.301) | <0.0001 | **0.458** | (0.400, | 0.517) | <0.0001 | **-0.230** | (-0.324, | -0.137) | <0.0001 |
| Long-term change^c^ | 0.013 | (-0.011, | 0.037) | 0.285 | -0.014 | (-0.030, | 0.002) | 0.090 | 0.027 | (-0.002, | 0.056) | 0.067 |
| **WOMAC-function** |  |  |  |  |  |  |  |  |  |  |  |  |
| Pre-operative score^a^ | **0.386** | (0.101, | 0.671) | 0.008 | 0.147 | (-0.051, | 0.345) | 0.146 | 0.239 | (-0.109, | 0.586) | 0.178 |
| Short-term change^b^ | **0. 201** | (0.133, | 0.269) | <0.0001 | **0.389** | (0.328, | 0.451) | <0.0001 | **-0.188** | (-0.280, | -0.096) | <0.0001 |
| Long-term change^c^ | 0.014 | (-0.008, | 0.037) | 0.219 | 0.005 | (-0.009, | 0.019) | 0.464 | 0.009 | (-0.017, | 0.036) | 0.495 |
| **Walking speed** |  |  |  |  |  |  |  |  |  |  |  |  |
| Pre-operative score^a^ | 0.073 | (-0.171, | 0.317) | 0.559 | 0.179 | (-0.003, | 0.360) | 0.054 | -0.106 | (-0.410, | 0.198) | 0.496 |
| Short-term change^b^ | **0.115** | (0.057, | 0.172) | <0.0001 | **0.150** | (0.099, | 0.201) | <0.0001 | -0.036 | (-0.112, | 0.041) | 0.364 |
| Long-term change^c^ | -0.017 | (-0.049, | 0.015) | 0.300 | **0.022** | (0.007, | 0.037) | 0.005 | **-0.039** | (-0.075, | -0.003) | 0.032 |

Coefficient with p-value<0.05 are highlighted in bold.

WOMAC-pain, function and walking speed are modelled as standardised outcomes. The regression coefficients are derived from random intercept and slope models adjusted for time of assessment parameterised as two linear splines (short-term changes and long-term changes) and age(centred on mean age: respectively 65y and 66y for primary and revision arthroplasties). The variances of random effects and correlation coefficients are not presented but are available on request.

a. Intercept: Estimated mean function or pain standardised score on the day of surgery.
b. Short-term changes: Estimated monthly mean change in pain or function standardised scores between the pre-operative and first post-operative assessments (~3-months).

c. Long-term changes: Estimated monthly mean change in pain or function standardised scores between the first and second post-operative assessments (~3 and ~12-months).

d. Difference between the revision and primary groups’ regression coefficients assessed with linear contrasts.
